# Effect of Asphalt Granulation on the Performance of Artificial Graphite Anode Materials for Lithium-Ion Batteries

**DOI:** 10.3390/ma17246224

**Published:** 2024-12-20

**Authors:** Cong Yang, Jun Gong, Zhao Jiang, Tiejun Lei, Qian Zheng, Qi Deng, Yejun Li

**Affiliations:** 1 College of Mechanical and Electrical Engineering, Hunan University of Science and Technology, Xiangtan 411201, China; congyang1122@163.com (C.Y.); dengqi0503@126.com (Q.D.); 2 Hunan Bobsun New Material Co., Ltd., Changsha 410199, China; jiangzhao@hnu.edu.cn (Z.J.); troubleltj@163.com (T.L.); 15513886901@163.com (Q.Z.); 3 School of Physics and School of Materials Science and Engineering, Central South University, Changsha 410083, China

**Keywords:** artificial graphite, anode materials, pitch, granulation, secondary particles

## Abstract

In order to investigate the effects of the softening point, the addition ratio, and the median particle size (D_v_50) of the asphalt on the performance of secondary particles of artificial graphite anode materials prepared by granulation, ten-kilogram orthogonal experiments were designed. D_v_50 and powder orientation index (OI) value of the prepared secondary particles of artificial graphite anode materials were employed as evaluation index, and the results of the orthogonal experiments were subjected to polarity analysis, analysis of variance (ANOVA), and multiple linear regression analysis. It is demonstrated that the addition ratio of the asphalt exerts the most pronounced influence on D_v_50 and powder OI value of secondary granular artificial graphite anode materials, followed by the softening point. Conversely, the median particle size D_v_50 of the asphalt exerts the least significant effect. With regard to the evaluation indices, the optimal granulation conditions were determined to be a softening point of 250 °C, an addition ratio of 11%, and D_v_50 of 2–3 μm of the asphalt. D_v_50 of the prepared secondary granular artificial graphite anode material was 17.91 μm, with an OI value of 1.91. The specific capacity of the first charging was 340.52 mAh/g, and the first columbic efficiency was 94.79%.

## 1. Introduction

Lithium-ion batteries have been employed extensively in many fields, offering a plethora of advantages, including high specific energy density, high operating voltage, the absence of a memory effect, and the benefit of being environmentally friendly and pollution-free [1]. As the use of lithium-ion batteries proliferates across an array of devices [2], there is a concomitant increase in the expectations placed upon these batteries in terms of the capacity, multiplication rate, and cycle stability. Lithium-ion batteries rely on the anode materials as a pivotal component in enhancing the overall performance, including the capacity, charging speed, and service life [3].

Since Sony introduced carbon materials instead of lithium metals as the anode electrodes in the first commercial lithium-ion battery [4], carbon materials (i.e., natural graphite [5], man-made graphite [6], carbon nanofibres [7], carbon nanotubes [8], and graphene [9], among others) have been widely studied as the anode electrodes for lithium-ion batteries. Currently, artificial graphite materials are the preferred materials for the anode materials of lithium-ion batteries [10]. In order to improve the performance of lithium-ion batteries, research has been carried out on the modification of the anode materials and the granulation process. The artificial graphite granulation process involves the formation of large particles through the bonding of crushed small particles of aggregate with a binder [11]. This is followed by the subsequent graphitisation of the particles, resulting in the production of secondary particles of artificial graphite anode materials. Fu Y [12] et al. prepared the secondary particulate graphite anode materials by co-pyrolysis of coking coal and graphite tailings. The results demonstrated that the coking coal pyrolysis product not only achieved lamellar reconstruction of the graphite tailings but also enhanced the anisotropy of the anode material. Wu [13] et al. prepared carbon-coated isotropic natural graphite by spray granulation utilising ultrafine graphite powders (a by-product of spherical natural graphite) as the raw materials. The sample displays a complete microstructure with isotropic characteristics, which effectively mitigate the local stress concentration that occurs during lithium-ion insertion/extraction while enhancing the electrical conductivity. Guo Mingcong [14] et al. utilised a homemade high-performance coal asphalt as a binder for the pelletising process and investigated the impact of asphalt addition on the performance of secondary particles of artificial graphite anode electrodes. They discovered that the optimal pelletising process was achieved with an asphalt addition of 8%, resulting in an enhanced high-rate charge/discharge performance. Jian Huang [15] et al. investigated the effect of asphalt-coated natural graphite with varying softening points on its structural and electrochemical characteristics. It was determined that the natural graphite coated with asphalt exhibiting a high softening point and a substantial concentration of Toluene Insoluble substance (TI) and Quinoline Insoluble substance (QI) components would result in the formation of a compact and inadequate carbon layer on the surface, with an enhanced cycling performance and reversible capacity. Zhang J [16] et al. prepared secondary particles of artificial graphite by wet granulation. It was observed that the rate performance of the secondary particles of artificial graphite was markedly enhanced in comparison to that of the original artificial graphite. Wang Jiwei [17] et al. examined the impact of utilising a secondary granulated graphite anode material on battery performance, with the preparation of square batteries incorporating both single and secondary granulated graphite anode materials. The findings indicated that the square batteries fabricated with the two anode materials exhibited reduced internal resistance and enhanced capacity retention in comparison to the square batteries prepared with the secondary particle anode material. The secondary particles of artificial graphite anode material can enhance the material isotropy and simultaneously reduce the migration distance of lithium ions within the anode materials. To a certain extent, this can enhance the electrical conductivity and mitigate the expansion issue observed in batteries subjected to prolonged cycling. The secondary particle anode materials prepared through the granulation process exhibit a low impedance, which is a notable advantage.

The majority of research conducted thus far on the granulation process has been mainly focused on single factors. In the context of the actual enterprise granulation process, a multitude of factors, including equipment conditions, process formulations, raw material properties, and other variables, must be considered. In light of the aforementioned research, the experimental conditions proposed in this paper are more closely aligned with the conditions observed in the actual enterprise high-volume granulation process, where we employed the pilotscale test equipment to conduct three-factor, three-level orthogonal experiments with needle raw coke as aggregate and asphalt as a binder. The primary analytical evaluation indices are the median particle size D_v_50 and the size of powder OI value of the secondary particles of artificial graphite electrode materials. The effects of the three parameters, namely the softening point, the addition ratio, and the median particle size D_v_50 of the asphalt, on the performance of secondary granular artificial graphite were investigated in the pelletising process through polar analysis, analysis of variance, and multiple linear regression analysis. This provides a theoretical basis for the design of the granulation process and serves as a reference for the mass production and preparation of secondary particles of artificial graphite in industry.

## 2. The Orthogonal Experimental Scheme and Procedure

### 2.1. Experimental Materials

The raw coke was prepared by crushing needle-shaped aggregates using an impact mill, resulting in a powder with a median particle size of 9.71 μm and a vibration density of 0.61 g/cm^3^.

Three distinct softening points of asphalt were selected for the binder, and their fundamental properties (softening point, coking value, quinoline insoluble substance, toluene insoluble substance, and ash) are presented in Table 1. The asphalt was pulverised by an airflow mill into three distinct specifications of asphalt powders with varying median particle sizes D_v_50, respectively. The median particle size D_v_50 of the three specifications was found to be 2.59 μm, 4.63 μm, and 6.42 μm, respectively.

### 2.2. Orthogonal Experimental Design

In this experiment, the softening point, the addition ratio, and the median particle size D_v_50 of the asphalt were selected as the main factors of the experiment (recorded as Factor A, Factor B, and Factor C, respectively). To ensure the reliability of the results, three levels were set for each factor, thus carrying out three-factor, three-level orthogonal experiments. The specific settings are detailed in Table 2.

### 2.3. Sample Preparation

**Figure 1:**
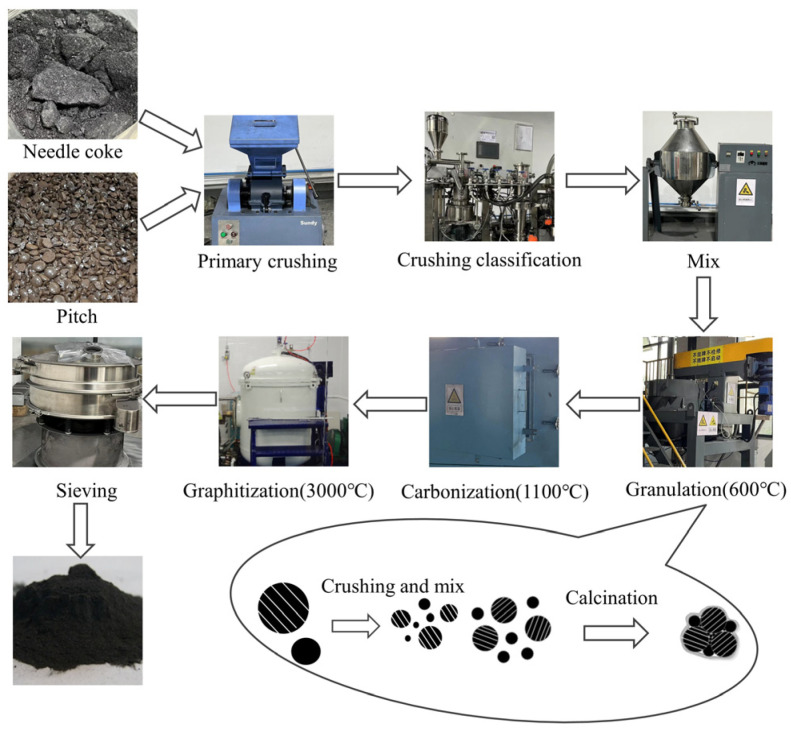
The preparation flow of the artificial graphite anode materials.

The preparation process of secondary granulated artificial graphite anode material is divided into five steps, i.e., material pretreatment, granulation, carbonisation, graphitisation, and screening, as illustrated in Figure 1. The material pretreatment involves crushing and classifying raw needle coke materials and asphalt auxiliary materials. The granulation process entails the mixing of the needle coke powder and asphalt in a specified proportion, followed by a heating reaction in high-temperature granulation equipment, resulting in the formation of a granulation mixture upon cooling. Subsequently, the material is carbonised and graphitised, after which the secondary particles of artificial graphite anode materials are sieved.

The objective of this study is to investigate the effects of the softening point, the addition ratio, and the median particle size D_v_50 of the asphalt on the properties of secondary granulated artificial graphite. The specific preparation process is outlined below:

(1) A total of 9 portions, each weighing 10 kg of raw needle coke powder, were prepared. In accordance with the orthogonal experimental table, distinct ratios of the asphalt were incorporated separately and blended in a double-cone mixer at a frequency of 50 Hz for a period of 2.5 h, ensuring comprehensive homogenisation.
(2) After introducing the mixture into the reaction kettle, setting the stirring paddle speed at 45 rpm under the protective atmosphere of nitrogen at 4 L/min, raising the temperature to 600 °C at the rate of 2 °C/min, and maintaining the temperature for 1.5 h, the granulation mixtures were obtained.
(3) The pelletised mixtures were placed in a box charcoal oven and subjected to a preliminary charring process at 1100 °C, with the objective of eliminating any residual volatile compounds. The pelletised mixtures exhibited a reduction in volatile compounds.
(4) The low volatile fraction granulation mixtures were subjected to high-temperature graphitisation in a graphitising furnace at 3000 °C. Subsequently, the secondary granulated artificial graphite anode materials were prepared.
(5) The prepared secondary particles of artificial graphite anode materials were subjected to a 325 mesh sieving process to remove the large particles.

The nine samples were designated as ZLB110-4%-1, ZLB110-11%-3, ZLB110-18%-2, ZLB180-4%-3, ZLB180-11%-2, ZLB180-18%-1, ZLB250-4%-2, ZLB250-11%-1, and ZLB250-18%-1, respectively, in accordance with the order of the orthogonal table of experiments. The first value represents the softening point of the asphalt, the second value represents the percentage of asphalt added, and the third value represents the range of the median particle size of the asphalt (1 for 2–3 μm, 2 for 4–5 μm, and 3 for 6–7 μm). In order to facilitate a comparative discussion of the impact of the granulation process on the properties of artificial graphite, the set of samples that have not undergone the granulation process will be designated as SYB.

### 2.4. The Process of Creating Button Batteries

The artificial graphite anode material, conductive agent (acetylene black SP), dispersant (carboxymethyl cellulose, CMC), and binder (styrene-butadiene rubber, SBR) were combined in distilled water with a mass ratio of 95:1:1.5:2.5. The slurry was then stirred thoroughly and evenly to create a uniform coating, which was subsequently applied to the copper foil using a coating machine. The coated foil was then subjected to a vacuum drying process at 120 °C for 12 h. Following this, the dried foil was rolled into round shapes with a diameter of 14 mm using a sheet punching machine. The cut pole piece was then subjected to a drying process at 110 °C for 3 h in a vacuum drying oven. Following this, the mass of the pole piece and the empty copper foil was determined, and the mass of the active substance was calculated to be 6.5 mg/cm^2^ in accordance with the proportioning.

In this study, a CR2032 model button-type lithium-ion battery was assembled in a glove box under an argon atmosphere. In the assembled lithium-ion battery, the counter electrode is a lithium wafer with a diameter of 15.4 mm, the diaphragm is composed of a Celgard 2325 polypropylene membrane sourced from the United States, and the electrolyte was 1 M LiPF6 (lithium hexafluorophosphate) in a solution of EC (ethylene carbonate), DMC (dimethyl carbonate) and EMC (ethyl methyl carbonate) in a volume ratio of 1:1:1.

### 2.5. Analytical Characterization

The particle size and vibration density of the samples were examined using a Bettersize 2000 laser particle sizer and a vibration density meter from Dandong Baxter (Dandong, China). The specific surface area of the samples was examined using a BSD-BET400 Specific Surface Adsorption Meter from Bayside (Beijing, China). The microstructure of the samples was characterised using a scanning electron microscope JSM-IT200 from Nippon Electron Corporation (Tokyo, Japan). The crystal structure of the artificial graphite anode materials was analysed using a Bruker D2 X-ray diffractometer (Karlsruhe, Germany). The analysis was conducted on the artificial graphite anode material. The electrochemical performance of the button-type lithium-ion battery was tested using a Xinwei CT-4008T-5V10ma-164 (Shenzhen, China) battery test system and a Princeton VersaSTAT 3A electrochemical workstation (Pennsylvania, PA, USA).

## 3. Analysis and Discussion of Experimental Results

The experiments were conducted in accordance with the orthogonal experimental design scheme outlined in Table 3. The results of the experiment were used to analyse the impact of each factor on the properties of secondary particles in artificial graphite materials. The median particle size D_v_50 and powder OI value size of the material were selected as the primary evaluation indices. Subsequently, polar analysis, analysis of variance, and multiple linear regression analysis were conducted.

### 3.1. Physical Property Analysis of Secondary Particles of Artificial Graphite

Table 4 illustrates the physical properties of single-particle artificial graphite anode material SYB and secondary-particle artificial graphite anode materials ZLB110-4%-1, ZLB110-11%-3, ZLB110-18%-2, ZLB180-4%-3, ZLB180-11%-2, ZLB180-18%-1, ZLB250-4%-2, ZLB250-11%-1, and ZLB250-18%-3. The granulation process involves the bonding of multiple small particles of needle coke aggregate into larger particles via asphalt binder. The larger particles exhibit a diminished surface area relative to the original smaller particles. Therefore, the asphalt serves not only as a binder but also as a surface modifier. During the granulation process, the asphalt fills the voids and imperfections on the surface of the particles, thereby creating a smoother surface. To a certain extent, this also results in a reduction in the specific surface area. Consequently, the specific surface area of secondary particles of artificial graphite is smaller than that of single particles of artificial graphite.

As illustrated in Table 4, the median particle size D_v_50 of the secondary particles of artificial graphite prepared by asphalt granulation is consistently larger than that of the single-particle artificial graphite SYB. A comparison of the three types of secondary particles of artificial graphite, namely ZLB110-4%-1, ZLB110-11%-3, and ZLB110-18%-2, reveals that the median particle size D_v_50 of the secondary particles of artificial graphite increases with the proportion of asphalt added, up to a softening point of 110 °C. The largest median particle size, D_v_50, is observed in ZLB110-18%-2, with an asphalt addition ratio of 18%. A comparison of the median grain size D_v_50 of the ZLB180-4%-3, ZLB180-11%-2, and ZLB180-18%-1 samples revealed that the largest median grain size D_v_50 was observed for the ZLB180-11%-2 sample, with an asphalt addition ratio of 11%. A comparison of ZLB250-4%-2, ZLB250-11%-1, and ZLB250-18%-3 revealed that the material with an 11% asphalt addition ratio exhibited the largest median grain size, D_v_50. It is hypothesised that when 18% of the asphalt with a softening point of 180 °C and 250 °C is added for pelletising, the excess asphalt does not bind the small particles of acicular raw coke aggregate, resulting in the formation of small particle coke. Subsequently, the 110 °C, 180 °C, and 250 °C softening point asphalt granulation group, which exhibited the greatest median particle size D_v_50 growth within the largest formula group, was selected for comparison. The median particle size D_v_50 of the ZLB250-11%-1 group ranged from 9.83 μm to 17 μm, representing a growth of 82.19%, which is larger than those of ZLB180-11%-2 (62.05%) and ZLB110-18%-2 (56.05%), indicating a pronounced granulation effect of the 250 °C softening point asphalt. This is due to the fact that Table 1 reveals that the residual carbon rate of the 250 °C softening point bitumen is 71.68%, which is the highest residual carbon rate among the three different softening point bitumens. In the artificial graphite pelletising process, the residual carbon of the bitumen generates large particles by shaping the small particles into a defined form. Consequently, the greater the residual carbon rate of the bituminous binder, the more favourable the granulation results. The median particle size D_v_50 of asphalt may have a negligible impact on the granulation effect. This is because the granulation process involves the melting and coking of asphalt. One of the critical factors in the granulation process is the presence of asphalt in a molten state, which facilitates adequate mixing and bonding. However, the median particle size of asphalt D_v_50 is not significantly correlated with the asphalt molten state.

The vibrational densities of the secondary particles of the artificial graphite, as presented in Table 4, are all less than those of the single-particle artificial graphite. This can be attributed to the presence of voids in the granulation and bonding process, which results in a greater volume of material after vibration in comparison to the single-particle material. Consequently, the vibration density is diminished.

### 3.2. XRD Analysis of Secondary Particles of Artificial Graphite

**Figure 2:**
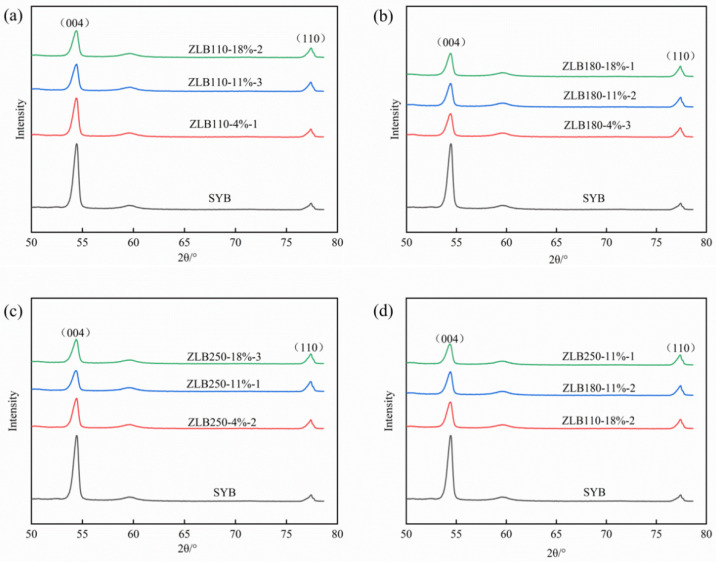
Illustration of X-ray diffraction (XRD) patterns of asphalt pelletised artificial graphite at temperatures of (**a**) 110 °C, (**b**) 180 °C, (**c**) 250 °C, and (**d**) at varying softening points.

Figure 2 illustrates the X-ray diffraction (XRD) pattern of the artificial graphite anode material.

As illustrated in the figure, the distinctive diffraction peaks of the (004) and (110) crystal planes of graphite were observed at approximately 54.4° and 77.3°, respectively, for the secondary particles of artificial graphite prepared via the granulation process [18]. These positions are essentially identical to those of the diffraction peaks of the single particles of artificial graphite prepared using an ungranulated process. This suggests that the granulation process does not alter the graphite crystal structure and that the prepared secondary particles of artificial graphites retain the graphite structure with favourable crystalline properties. The figure illustrates that the intensity of the (004) diffraction peaks differs between samples prepared after the granulation of the asphalt with varying softening points and addition ratios. Figure 2d illustrates that the intensity of the (004) diffraction peak decreases with an increase in the softening point of the asphalt added by pelleting. In the experimental group of the asphalt granulation at a softening point of 110 °C, as illustrated in Figure 2a, the diffraction peak intensity of the (004) diffraction pattern is lowest for the sample with an addition of 18%. In the experimental group of the asphalt granulation at 180 °C and 250 °C, the lowest diffraction peak intensity of (004) was observed with an addition of 11%, as shown in Figure 2b,c. This may be associated with the residual carbon of the asphalt. It can be observed that an increase in the residual carbon is accompanied by a reduction in the diffraction intensity of the corresponding samples prepared.

The OI value indicates the orientation of the materials [19], which is typically expressed as the ratio of I(004)/I(110). A lower OI value indicates greater isotropy of the material, faster diffusion of lithium ions within the anode material, and a reduced expansion rate. The OI values of the various artificial graphite samples are presented in Table 5.

As evidenced in Table 5, the OI value of secondary particles of artificial graphite is markedly lower than that of single particles of artificial graphite. This suggests that the isotropy of secondary particles of artificial graphite is superior to that of single particles. The OI value of ZLB250-11%-1 secondary particles artificial graphite is the lowest, with a value of 1.91, which is 76.27% lower than the OI value of single particles artificial graphite. Furthermore, Table 4 reveals that the size of the OI value is inversely proportional to the median particle size of secondary granular artificial graphite. In samples exhibiting a favourable granulation effect, a correlation can be observed between the median particle size D_v_50 and the OI value, whereby an increase in D_v_50 is associated with a reduction in OI value and an improvement in isotropy.

### 3.3. A Micro-Morphological Analysis of Secondary Particles of Artificial Graphite

**Figure 3:**
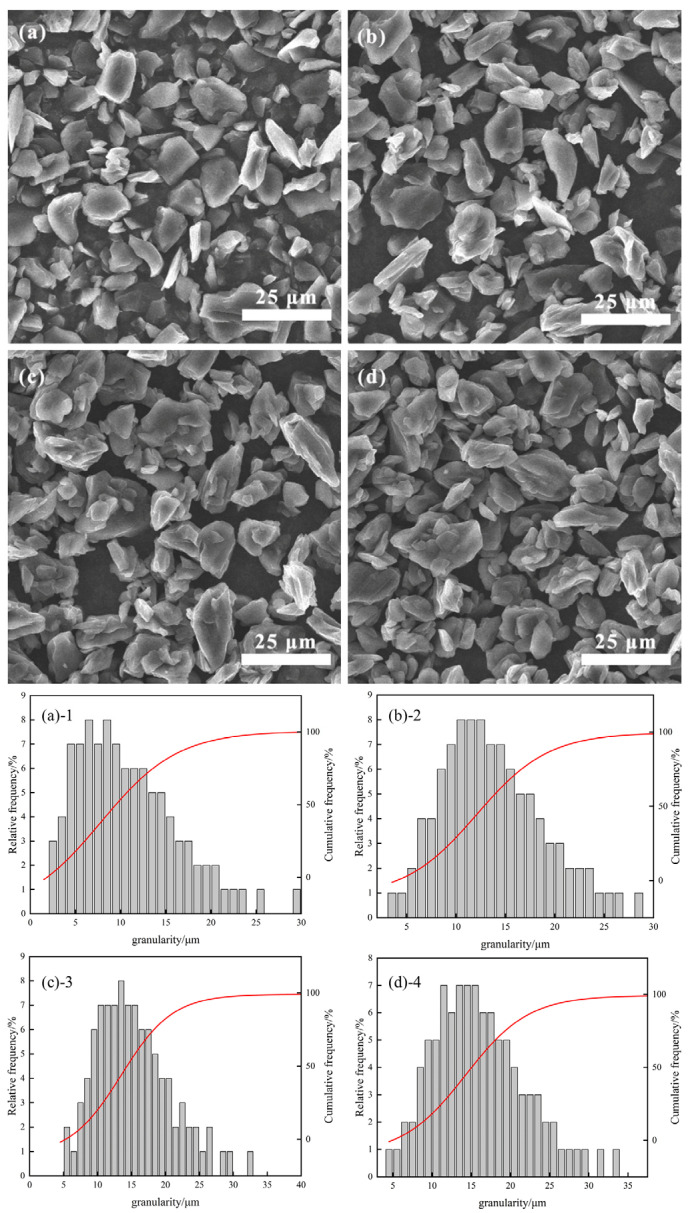
(**a**), (**(a)-1**) Single particle 110 °C asphalt added (**b**), (**(b)-2**) 4%, (**c**), (**(c)-3**) 11%, and (**d**), (**(d)-4**) 18%.

**Figure 4:**
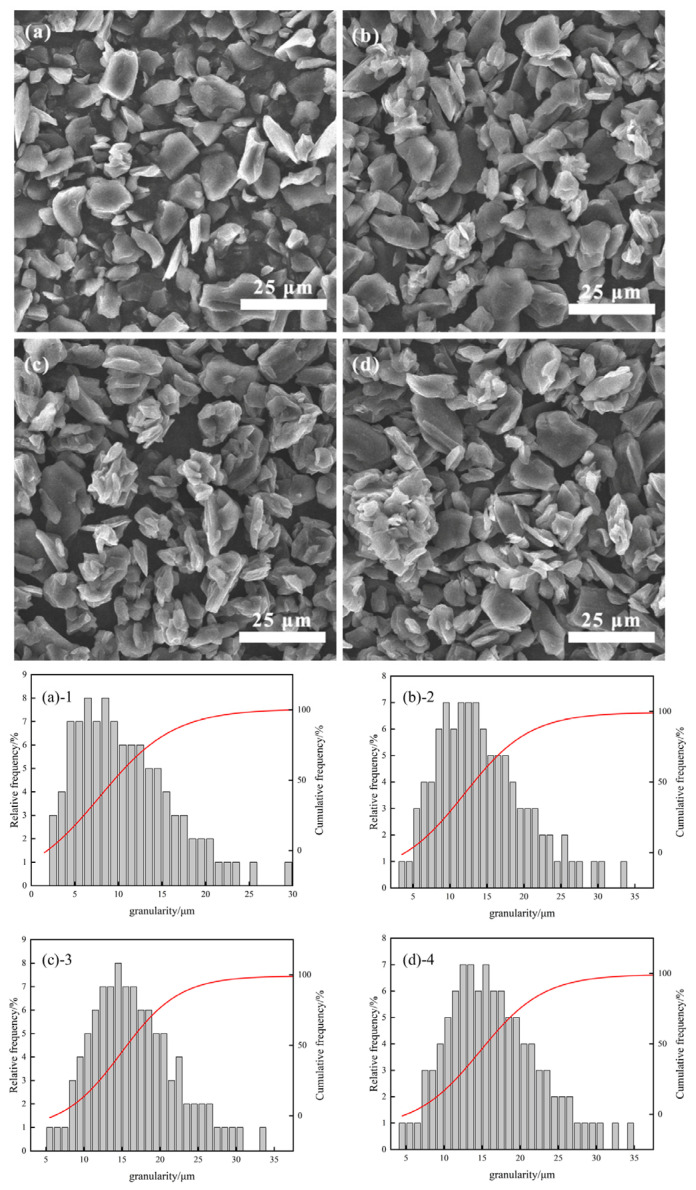
(**a**), (**(a)-1**) Single particle 180 °C asphalt added (**b**), (**(b)-1**) 4%, (**c**), (**(c)-1**) 11%, and (**d**), (**(d)-1**) 18%.

**Figure 5:**
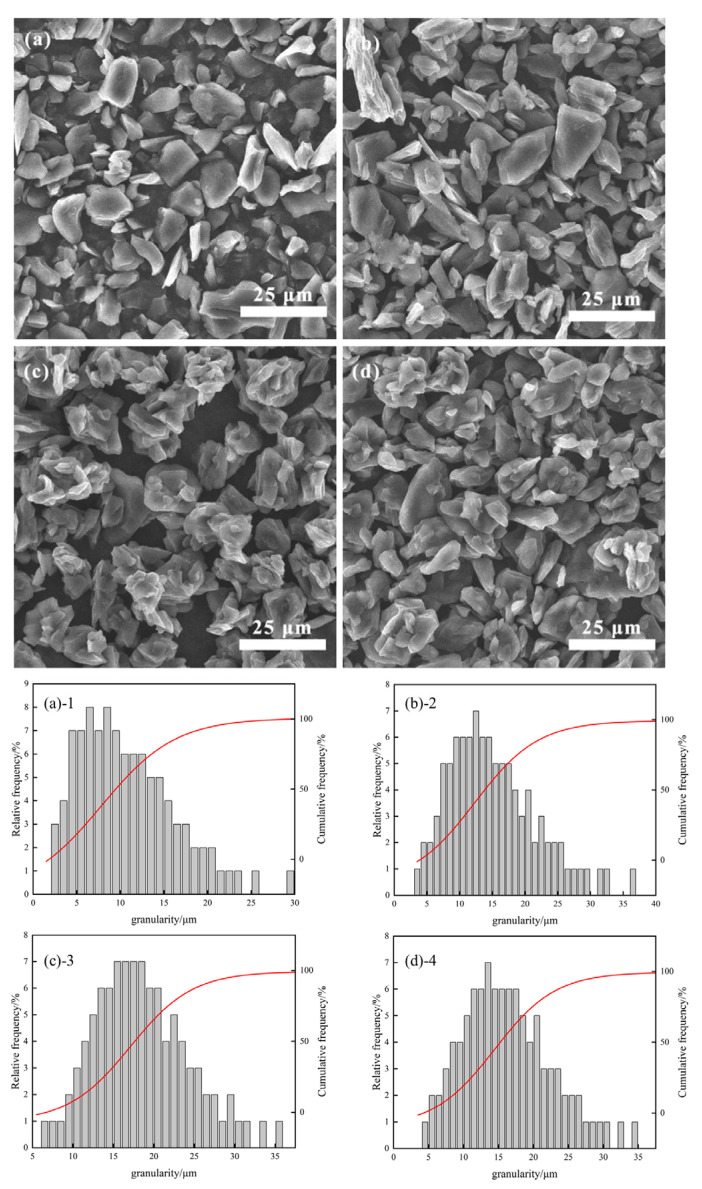
(**a**), (**(a)-1**) Single particle 250 °C asphalt added (**b**), (**(b)-1**) 4%, (**c**), (**(c)-1**) 11%, and (**d**), (**(d)-1**) 18%.

Figure 3, Figure 4 and Figure 5 illustrate scanning electron microscopy (SEM) images and particle size histograms of secondary particles of artificial graphite prepared by asphalt granulation at temperatures of 110 °C, 180 °C, and 250 °C, which correspond to the softening points of the asphalt. The images show single particles of artificial graphite (SYB)(a), as well as asphalt additions of (b) 4%, (c) 11% and (d) 18%, respectively. The corresponding particle size histograms are (a)-1, (b)-2, (c)-3 and (d)-4.

As illustrated in the figure, the morphology of SYB single-particle artificial graphite anode material is typically lamellar. However, the microscopic morphology of the secondary particles of artificial graphite anode material prepared through the granulation process differs from lamellar. Additionally, the secondary particles bonded by multiple small particles will appear [20]. As can be observed in Figure 3, the formation of secondary particles is relatively limited when 4% of asphalt granulation is added. However, the addition of 18% asphalt granulation results in a notable increase in the number of secondary particles. In Figure 4, the optimal granulation is achieved with the addition of 11% asphalt granulation. In Figure 5, the secondary particles formed with the addition of 11% asphalt granulation exhibit a more uniform distribution. However, the adhesion of multiple secondary particles results in the formation of larger particles when 18% of asphalt granulation is added, with the inhomogeneity of the sample. The micro-morphological analysis of the material indicates that the proportion of asphalt added during the granulation process is within an optimal range. The particle size histograms corresponding to the SEM plots demonstrate that the number of large particles produced by granulation using a 110 °C softening point bitumen increases with the additional ratio of bitumen. Furthermore, the highest number of large particles is produced by granulation using a 180 °C and 250 °C softening point bitumen at an addition ratio of 11%. At the same asphalt addition ratio, the use of 250 °C softening point asphalt for granulation results in the production of a greater number of large particles, which further supports the assertion that the use of 250 °C softening point asphalt granulation is a superior approach.

### 3.4. Polar Analysis of Median Particle Size D_v_50 and OI Values

In this study, the median particle size D_v_50 and powder OI value of artificial graphite are employed as evaluation indices for the assessment of granulation advantages and disadvantages. It can be observed that within a certain range, an increase in the median particle size D_v_50 and a decrease in the OI value are indicative of an enhanced granulation effect.

Extreme variance analysis was employed to investigate the impact of three key factors, namely the softening point of asphalt, the addition ratio, and the median particle size D_v_50, on the median particle size D_v_50 and OI value of the secondary granular artificial graphite. In this context, *K_i_* represents the sum of the values of the evaluation indexes under each level of each factor, while *k_i_* denotes the mean value of the values of the evaluation indexes under each level of each factor. Finally, *R* signifies the extreme deviation of the value of *k_i_*. The results of the polar analysis, which demonstrate the influence of each factor on the median particle size D_v_50 and OI value of secondary particles of artificial graphite, are presented in Table 6.

The extent to which different factors exert influence on each evaluation indicator can be elucidated through the utilisation of the range analysis. The greater the range is, the more significant the impact of the factor on the corresponding evaluation indicator [21]. Table 6 reveals a correlation between the median particle size D_v_50 of secondary granular artificial graphite, the softening point of asphalt, the addition proportion, and the median particle size D_v_50, where the ranges of the median particle sizes of the three factors were 1.64 μm, 2.58 μm, and 0.89 μm, respectively. Among the three factors, the addition proportion of asphalt exhibited the largest range, followed by the softening point of asphalt and the median particle size D_v_50 of asphalt (B > A > C). This suggests that the proportion of asphalt addition is the most significant factor influencing the median particle size D_v_50 of secondary granular artificial graphite. With regard to the OI value of secondary artificial graphite, the greatest range is observed in the case of the asphalt addition ratio, while the smallest range is found in the case of the asphalt median particle size D_v_50. In terms of the influence degree of the OI value, the order is thus B > A > C. With regard to the median particle size D_v_50 and the OI value, the degree of influence exerted by each factor is comparable.

**Figure 6:**
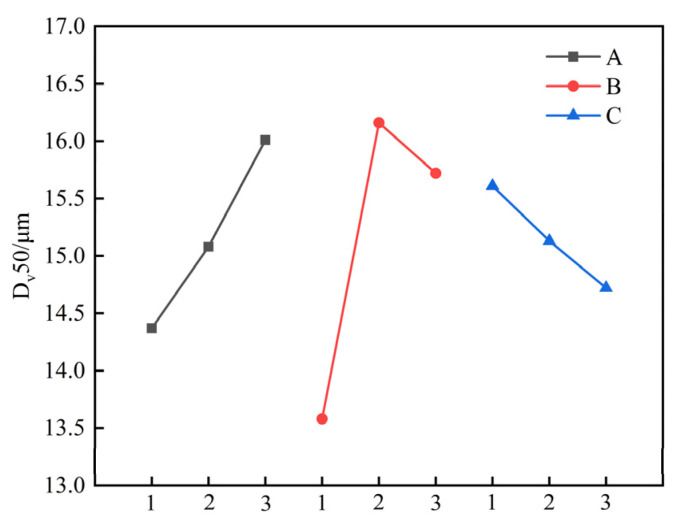
D_v_50 as a function of different factors at each level.

**Figure 7:**
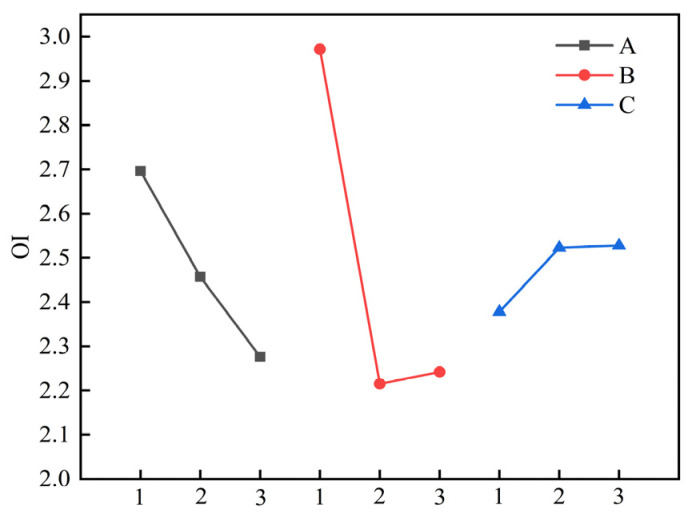
OI as a function of different factors at each level.

In order to gain a more intuitive understanding of the trend in the median particle size D_v_50 and OI value of secondary particles, artificial graphite, and the influence of each factor, the corresponding intensity curves were plotted for different levels of each factor in Figure 6 and Figure 7.

As illustrated in Figure 6, the median particle size D_v_50 of the secondary particles of artificial graphite demonstrates an increase with the softening point of the bitumen introduced through pelletisation. This increase occurs initially and then reverses with the rise in the proportion of the added asphalt before declining with the rise in the median particle size D_v_50. The optimal combination of factors, as determined by the median particle size D_v_50 as the evaluation index, is A_3_B_2_C_1_.

As illustrated in Figure 7, the trend of the OI value of secondary particles of artificial graphite exhibits an inverse relationship with that of D_v_50. The size of the OI value demonstrates a decrease with an increase in the softening point of the added asphalt, followed by an increase and subsequent decrease with an increase in the addition ratio and an increase with an increase in the median particle size. This is due to the inverse relationship between the OI value of secondary artificial graphite and the median particle size D_v_50. When the OI value is employed as the evaluation index, the optimal combination of each factor is identified as A_3_B_2_C_1_, which is identical to that determined by D_v_50.

### 3.5. ANOVA for Median Particle Size D_v_50 and OI Values

The extreme variance analysis method is capable of responding intuitively to the level differences of factors. However, it is unable to determine the weight size of each factor directly. To further analyse the degree of influence of the three factors, namely the softening point, addition ratio, and median particle size D_v_50 of the asphalt, on the median particle size D_v_50 and OI value of secondary granular artificial graphite, the results of the orthogonal experiments were subjected to analysis of variance (ANOVA).

The results of the ANOVA are presented in Table 7. A *p*-value of less than 0.05 indicates that the effects are significant, whereas a value greater than 0.05 indicates that they are not significant. The table reveals that the proportion of asphalt addition exerts a significant influence on the OI value of secondary particles of artificial graphite. The larger the F value, the greater the impact of the influencing factors on the evaluation index [22]. When the median particle size D_v_50 and OI value are employed as evaluation indices, the results indicate that the factor with the greatest influence on the granulation effect is the proportion of asphalt added, followed by the softening point of asphalt. The factor with the least influence is the median particle size D_v_50 of asphalt, thus with an order of F_B_ > F_A_ > F_C_.

### 3.6. A Multiple Linear Regression Analysis Was Conducted on the Median Particle Size D_v_50 and OI Values

It is important to note that the pelleting process itself involves a constant alteration in the formulation, which makes it challenging to obtain precise results solely through polar analysis and ANOVA. Consequently, regression analysis must also be conducted on the experimental variables to investigate the correlation between the softening point of asphalt introduced by pelletising, the addition ratio and the median particle size D_v_50 of the asphalt, the median particle size D_v_50 and OI values of secondary granulated artificial graphite.

The initial step involved the modelling of multiple regression equations [23].

$$y=\beta_{0}+\beta_{1}x_{1}+\cdots +\beta_{m}x_{m}$$

In a regression analysis, the independent variables *x*_1_, *x*_2_, *x*_3_, etc. are used to predict the dependent variable y, with *β*_0_ representing the regression constant and *β*_1_, *β*_2_, etc. representing the regression coefficients. The independent variables are defined as follows: asphalt softening point A, asphalt addition ratio B, and asphalt median particle size C.

The use of statistical analysis software, specifically SPSS27, facilitated the execution of multiple linear regression analyses on three key variables: asphalt softening point A, asphalt addition ratio B, and asphalt median particle size C. The objective was to establish a multiple linear regression model for the median particle size D_v_50 and OI value of the secondary granular artificial graphite. The results of the multiple linear regression analysis for the median particle size D_v_50 are presented in Table 8.

The correlation coefficient R^2^ of the regression model with median particle size D_v_50 as the dependent variable was 0.69, indicating that all the changes in the independent variables explained 69% of the changes in the dependent variable. In the multivariate linear test for the model, it was found that the Variance Inflation Factors (VIF) were all less than 5, indicating that there is no issue with the independent variables being collinear. Additionally, the D-W value was 2.83, which falls within the range of 0–4, suggesting that the independent variables adhere to the assumption of independence. The model exhibits no autocorrelation, and there is no correlation between the sample data, which suggests that the present model is superior to the previous model [24].

Table 8 presents the regression coefficients, which are 12.26, 0.82, 1.07, and −0.44. This information allows us to construct the multiple linear regression model as follows:

$$y=12.26+0.82x_{1}+1.07x_{2}-0.44x_{3}$$

**Figure 8:**
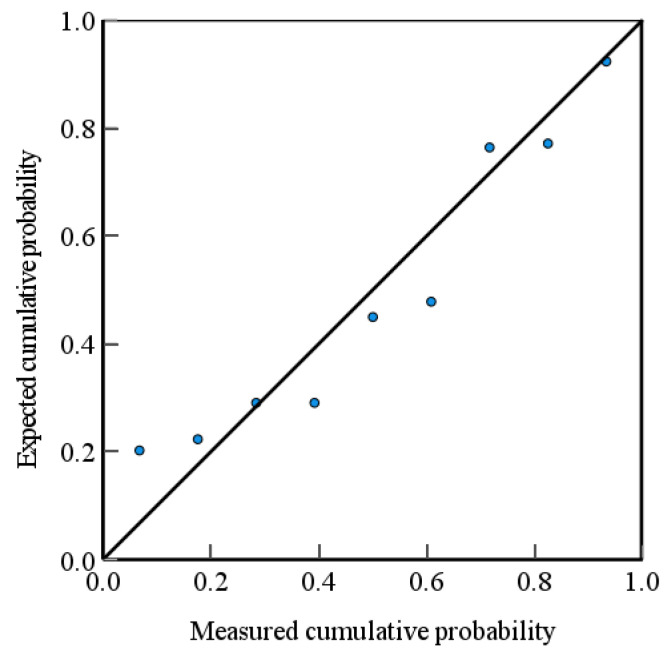
D_v_50 normal distribution p-p plot.

The regression theory analysis indicates a positive correlation between the softening point and the addition proportion of the asphalt with the median particle size D_v_50 of secondary granular artificial graphite. Conversely, the median particle size D_v_50 of asphalt is negatively correlated with the median particle size D_v_50. The asphalt addition ratio corresponds to the lowest *p*-value, indicating that this factor exerts the greatest influence on the median particle size of secondary granular artificial graphite. Figure 8 illustrates the normally distributed P-P plot resulting from the multiple linear regression analysis of median particle size D_v_50 [25].

Furthermore, the residuals of the regression model were subjected to the S-W test to ascertain whether they exhibited a normal distribution. A *p*-value exceeding 0.05 signifies that the data conformed to a normal distribution. The S-W test table is presented below for reference.

As evidenced by Table 9, the probability value of 0.23 is greater than 0.05, indicating that the residuals of the regression model adhere to a normal distribution. This signifies that the linear regression has reached the normality condition and that the model is capable of accurate and reliable prediction.

The results of the multiple linear regression on the OI values are presented in Table 10. As shown, the correlation coefficient R^2^ of the regression model derived using the OI values as the dependent variable is 0.76, indicating that 76% of the dependent variable can be explained by the current change in the independent variables. In the multivariate linear test of the model, it was found that all the VIF values were less than 5, indicating that the variables did not have covariance problems with each other. Furthermore, the D-W value was 2.83, which was located in the range of 0–4. It could be considered that there was no autocorrelation in the model of the independent variables. This is consistent with the independence of the variables and indicates that there is no correlation between the sample data. Consequently, the model is deemed to be better.

Table 10 presents the regression coefficiencies, which are 3.48, −0.21, −0.37, and 0.08. The multiple regression equation can thus be modelled as follows:

$$y=3.48-0.21x_{1}-0.37x_{2}+0.08x_{3}$$

**Figure 9:**
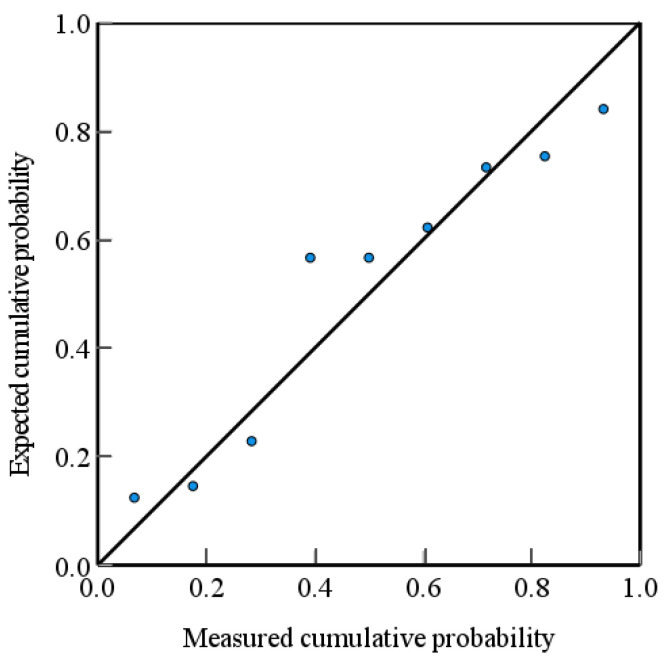
A p-p plot of the normal distribution of OI values.

A reduction in the OI value is indicative of an enhanced granulation effect. The regression coefficient analysis indicates that the softening point, the addition proportion of the asphalt, and the OI value of secondary particles of artificial graphite are negatively affected. In other words, the higher the softening point of asphalt and the addition proportion are, the smaller the OI value and the better the granulation effect is. It can be seen that the asphalt addition ratio (*p* = 0.02) is less than 0.05, which indicates that the asphalt addition ratio has a significant effect on the OI value. The median particle size D_v_50 of the asphalt has a slight positive effect on the OI value, indicating that as the D_v_50 increases, the OI value also increases, and the pelletising effect deteriorates. The results of the multiple linear regression analysis of the OI value are consistent with the results of the multiple linear regression analysis of the D_v_50, further indicating that the asphalt addition ratio has the greatest influence on the pelletising effect and the performance of the artificial graphite material. It is also shown that when determining the softening point of the asphalt and the median particle size D_v_50, it is possible to increase the asphalt addition ratio within a certain range in order to enhance the granulation effect. The normal distribution of the multiple linear regression analysis with the OI value as the dependent variable is illustrated in Figure 9.

As shown in Figure 9, the scatter points are distributed along or in close proximity to the diagonal line, which aligns with the anticipated relationship. Table 11 presents the S-W test table of the residuals of the regression model. The resulting *p*-value of 0.22 is greater than 0.05, indicating that the residuals of the regression model follow a normal distribution. This implies that the model predictions are accurate and reliable.

### 3.7. Electrochemical Testing of Secondary Particles of Artificial Graphite

To ascertain whether the optimisation of the parameters obtained from the aforementioned analytical methods yielded the anticipated enhancements, electrochemical tests were conducted for comparison. The control group was composed of SYB single-particle artificial graphite, while the optimised group was constituted by ZLB250-11%-1, a secondary-particle artificial graphite prepared from the best granulation combination.

**Figure 10:**
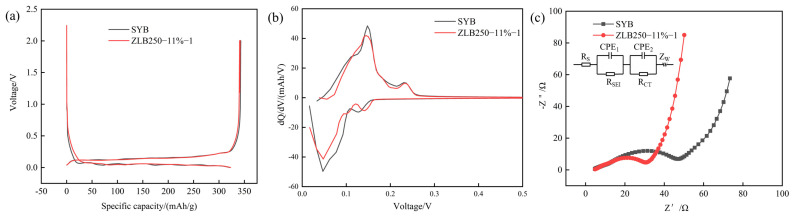
Electrochemical performance curve of artificial graphite anode materials (**a**) charge and discharge curves, (**b**) dQ/dV profiles of graphite, (**c**) AC impedance curves.

Figure 10 illustrates the distinct electrochemical performance curves of SYB and ZLB250-11%-1. Figure 10a shows that the charge-discharge curves of both single-particle and secondary-particle artificial graphite anode materials exhibit the characteristic U-shaped charge-discharge profile of graphite [26], with a discernible charge-discharge plateau. This suggests that the pelleting process does not disrupt the crystal structure of graphite and enables the normal de-embedded lithium reaction.

The particular initial charge/discharge test is illustrated in Table 12, and the initial charge/discharge specific capacity data selects the middle value of three button batteries. The initial charge-specific capacity and initial coulombic efficiency of ZLB250-11%-1 are 340.52 mAh/g and 94.79%, respectively. In comparison with SYB, the Coulomb efficiency exhibited an increase of 1.21%, from 93.58% to 94.79%. Conversely, the specific capacity demonstrated a slight decline. This is due to the necessity of incorporating asphalt binder into the granulation process, resulting in residual carbon. The specific capacity of the asphalt residual carbon is lower than that of artificial graphite. Consequently, the specific capacity of the obtained materials is lowered. Furthermore, the pitch modification will induce surface pore defects, thus resulting in a reduction in the specific surface area of the secondary particles of artificial graphite anode material. This, in turn, reduces the irreversible reaction, thereby increasing the first columbic efficiency.

As illustrated in Figure 10b, the dQ/dV curves of SYB and ZLB250-11%-1 exhibit notable similarities. The oxidation peak, which corresponds to the detachment of lithium ions from graphite, occurs between 0.1 and 0.2 volts, while the reduction peak, which corresponds to the embedding of lithium ions into the graphite layer, occurs between 0 and 0.1 volts [27]. A further comparison of the area enclosed by the dQ/dV curve and the horizontal coordinate reveals that the area of ZLB250-11%-1 is smaller than that of SYB. This suggests that the charge/discharge capacity of the secondary granular artificial graphite ZLB250-11%-1 is smaller than that of the single-granular artificial graphite SYB, which is in agreement with the results of the specific capacity test.

Figure 10c illustrates the AC impedance curves of SYB and ZLB250-11%-1. The AC impedance curve of the artificial graphite anode material is characterised by a semicircular shape in the high-frequency region and a linear trend in the low-frequency region [28]. The corresponding equivalent circuit diagram is illustrated in the upper left corner of the figure. In the high-frequency region, one or two semicircles are typically observed, which correspond to the formation of the SEI (R_SEI_) and the charge transfer resistance R_CT_, respectively. In contrast, the inclined straight line in the low-frequency region represents the Warburg impedance Z_W_ for lithium ion diffusion [29]. Furthermore, the intersection of the high-frequency region with the Z’ axis represents the intrinsic resistance generated by the electrolyte, diaphragm, and collector, which is denoted as R_S_.

The figure illustrates that the R_CT_ of ZLB250-11%-1 secondary granulated artificial graphite is 26.28 Ω, which is considerably smaller than the R_CT_ of SYB single granulated artificial graphite, which is 42.86 Ω. This suggests that the charge transfer impedance of ZLB250-11%-1 is smaller than that of SYB. Additionally, there is a notable increase in the slope of the slash line in the low-frequency region, indicating that the lithium-ion diffusion impedance becomes smaller. This suggests that the granulation process may have the potential to significantly reduce the impedance of artificial graphite anode materials.

## 4. Conclusions

(1) Secondary particles of artificial graphite anode materials with varying structures and electrochemical properties were prepared by pelletising with the asphalt of different softening points, addition ratios, and median particle size D_v_50.
(2) In terms of physical properties, the median particle size D_v_50 of the secondary artificial graphite will be larger than that of single artificial graphite. Conversely, the OI value is inversely proportional to the median particle size D_v_50. The median particle size D_v_50 and OI value were employed as evaluation indices for polar analysis, analysis of variance, and multiple linear regression analysis. The order of influence of each factor was determined to be asphalt addition ratio, softening point, and median particle size D_v_50. The optimal granulation ratio is determined to be the asphalt with a softening point of 250 °C, an addition of 11%, and a median particle size D_v_50 of 2–3 μm.
(3) A comparison of electrochemical tests between single and secondary granular artificial graphite reveals that the R_CT_ of ZLB250-11%-1 secondary granular artificial graphite is 26.28 Ω, while the R_CT_ of SYB single granular artificial graphite is 42.86 Ω. It can be observed that the charge transfer resistance of secondary granular artificial graphite is smaller than that of single granular artificial graphite. However, the first specific capacity is slightly lower than that of single granular artificial graphite. The first charge-specific capacity and Coulombic efficiency of the secondary granular artificial graphite ZLB250-11%-1, prepared with the optimal granulation ratio (250 °C softening point bitumen, 11% addition ratio, 2–3 μm median particle size D_v_50), were 340.52 mAh/g and 94.79%, respectively.
(4) In comparison to single-particle artificial graphite, the low impedance of secondary-particle artificial graphite anode material is advantageous due to the reduction in OI value resulting from the pelletising process. This leads to an increase in material isotropy and a greater number of migration channels for lithium ions with shorter transport paths.

## Figures and Tables

**Table 1:** Fundamental characteristics of pitch binders.

Sample	t (sp)/°C	ω (cv)/%	ω (QI)/%	ω (TI)/%	ω (a)/%
L-110	110	36.77	0.75	0.02	0.01
L-180	180	58.75	0.77	3.20	0.01
L-250	250	71.68	0.23	12.17	0.01

**Table 2:** Orthogonal experiment factor level table.

Factor Level	A (Softening Point)/°C	B (Addition Ratio)/%	C (Median Diameter)/μm
1	110	4	2.59
2	180	11	4.63
3	250	18	6.42

**Table 3:** Orthogonal experiment table.

Number	Influencing Factor		
	A	B	C
1	110	4	2.59
2	110	11	6.42
3	110	18	4.63
4	180	4	6.42
5	180	11	4.63
6	180	18	2.59
7	250	4	4.63
8	250	11	2.59
9	250	18	6.42

**Table 4:** Physical properties of the artificial graphite anode material.

Sample	D_v_50/μm	Tap Density/(g/cm^3^)	Specific Surface Area/(m^2^/g)
SYB	9.83	1.25	1.12
ZLB110-4%-1	13.13	1.04	0.78
ZLB110-11%-3	14.64	1.05	0.59
ZLB110-18%-2	15.34	1.09	0.40
ZLB180-4%-3	13.51	1.04	0.78
ZLB180-11%-2	15.93	1.12	0.53
ZLB180-18%-1	15.79	1.11	0.44
ZLB250-4%-2	14.11	1.05	0.78
ZLB250-11%-1	17.91	1.20	0.51
ZLB250-18%-3	16.02	1.16	0.45

**Table 5:** The OI values of the prepared artificial graphite anode materials.

Sample	OI
SYB	8.05
ZLB110-4%-1	3.02
ZLB110-11%-3	2.56
ZLB110-18%-2	2.50
ZLB180-4%-3	2.99
ZLB180-11%-2	2.17
ZLB180-18%-1	2.20
ZLB250-4%-2	2.89
ZLB250-11%-1	1.91
ZLB250-18%-3	2.02

**Table 6:** Range analysis results of D_v_50 and OI.

Evaluation Index	Analysis Index	A	B	C
D_v_50/μm	*K_1_*	43.11	40.75	46.83
	*K_2_*	45.23	48.48	45.38
	*K_3_*	48.04	47.15	44.17
	*k_1_*	14.37	13.58	15.61
	*k_2_*	15.08	16.16	15.13
	*k_3_*	16.01	15.72	14.72
	*R*	1.64	2.58	0.89
OI	*K_1_*	8.08	8.92	7.13
	*K_2_*	7.37	6.65	7.57
	*K_3_*	6.83	6.73	7.58
	*k_1_*	2.69	2.97	2.38
	*k_2_*	2.46	2.22	2.52
	*k_3_*	2.28	2.24	2.53
	*R*	0.42	0.75	0.15

**Table 7:** Anova results for D_v_50 and OI values.

Evaluation Index	Divisor	Deviation Sum of Squares Q	Degree of Freedom U	Mean Square M	F	Significance *p*	Partial Eta Squared
D_v_50/μm	A	4.08	2.00	2.04	4.56	0.18	0.82
	B	11.39	2.00	5.69	12.74	0.07	0.93
	C	1.18	2.00	0.59	1.32	0.43	0.57
	Error	0.89	2.00	0.45			
OI	A	0.27	2.00	0.13	7.88	0.11	0.89
	B	1.11	2.00	0.55	32.66	0.03	0.97
	C	0.04	2.00	0.02	1.29	0.44	0.56
	Error	0.03	2.00	0.02			

**Table 8:** D_v_50 linear regression analysis table.

	Normalized Coefficiency		Standardization Coefficiency			
	Regression Coefficiency B	Standard Error	β	t	*p*	VIF
Constant	12.26	1.52		8.06	<0.001	
A	0.82	0.43	0.48	1.92	0.11	1.00
B	1.07	0.43	0.62	2.49	0.06	1.00
C	−0.44	0.43	−0.26	−1.04	0.35	1.00
R^2^	0.69					
D-W	2.83					

**Table 9:** D_v_50 S-W test result table.

	Statistics	Degree of Freedom	Significance *p*
Unstandardized Residual	0.89	9.00	0.23
Standardized Residual	0.89	9.00	0.23

**Table 10:** The OI values linear regression analysis table.

	Normalized Coefficiency		Standardization Coefficiency			
	Regression Coefficiency B	Standard Error	β	t	*p*	VIF
Constant	3.48	0.39		9.00	<0.001	
A	−0.21	0.11	-0.43	−1.94	0.11	1.00
B	−0.37	0.11	-0.74	−3.36	0.02	1.00
C	0.08	0.11	0.15	0.69	0.52	1.00
R^2^	0.76					
D-W	2.83					

**Table 11:** OI values S-W test results table.

	Statistics	Degree of Freedom	Significance *p*
Unstandardized Residual	0.89	9.00	0.22
Standardized Residual	0.89	9.00	0.22

**Table 12:** First charge and discharge test results of artificial graphite anode materials.

Sample	Specific Capacity/(mAh/g)			Coulomb Efficiency/%
	Electric Discharge	Charging	Irreversible	
SYB-1	342.78	320.75	22.03	93.57%
SYB-2	342.88	320.87	22.01	93.58%
SYB-3	343.06	321.12	21.94	93.60%
ZLB250-11%-1-1	340.04	322.16	17.88	94.74%
ZLB250-11%-1-2	340.52	322.78	17.74	94.79%
ZLB250-11%-1-3	341.16	323.49	17.67	94.82%

## Data Availability

The original contributions presented in this study are included in the article. Further inquiries can be directed to the corresponding authors.
